# A Treatise on the More Obscure Affections of the Brain, on Which the Nature and Successful Treatment of Many Chronic Diseases Depend. Being the Gulstonian Lectures, Delivered at the College of Physicians, in May, 1835

**Published:** 1836-07

**Authors:** 


### Art. VI.

*A Treatise on the more obscure Affections of the Brain, on which the Nature and successful Treatment of many Chronic Diseases depend. Being the Gulstonian Lectures, delivered at the College of Physicians, in May,* 1835. By A. P. W. Philip, m.d., f.r.s. l. & e. Fellow of the Royal Colleges of Physicians of London and Edinburgh, &c.—*London,* 1835. Pp. 140.

The influence of the nervous system on the functions of other parts has been a subject of enquiry since the earliest period of anatomical research. The path of this enquiry has been beset with many difficulties, some of them necessarily connected with the nature of the subject, and others arising from the faultiness of the method employed in the investigation. Amongst the former may be classed the intricacy and minuteness of structure of the different parts of the nervous system, and the peculiarity of its functions; the faultiness of method consists chiefly in the disposition which men have to generalize their knowledge, by extending to the explanation of new facts principles with which they are familiar, while they neglect to enquire whether the analogy between the new facts and those previously observed be so strict as to warrant such extension.

The mass of the nervous system, in the higher animals, consists in a great degree of cellular and vascular tissues; the nervous substance, properly so called, bearing a small proportion to the general volume. In a paper by Dr. Macartney, published in the third volume of the Transactions of the British Association, that eminent physiologist has shown that, not only in the nervous trunks, but even in the brain, the greater part of its volume is formed by an extremely fine and delicate membrane, which pervades all its parts, and gives a covering and support to the minutest portions of nervous substance which enter into the composition of that organ. Dr. Macartney believes that the nervous substance, if it could, in the living state, be separated from all the tissues with which it is ordinarily combined, would be altogether invisible; and illustrates this position by observing that, in the human eye, the pigment of the choroid is seen distinct and unchanged in colour through the retina, as through the transparent humours of the eye; the human retina affording, perhaps, the best example of nervous substance, little accompanied by accessory tissues. In the other parts of the frame, in the skin or mucous membrane, for instance, if we attempt to trace the nervous filaments to their origins in those parts, we soon reach a point beyond which we can no longer distinguish separate filaments, or portions of nervous substance, clothed by cellular membrane. The filaments, as far as we can trace them, appear to have a plexiform arrangement; but we have reason to believe that there is not a single point on the surfaces we have mentioned which does not possess a portion of the nervous substance, and a mode of sensibility necessarily connected with its organization in that point. The nervous substance may be called the sentient substance, as being that tissue in which inheres the power of feeling the influence which various substances or circumstances are capable of exerting on the living structure. As there is no part in the living animal which does not possess a sensibility or mode of feeling of one kind or other, it is a legitimate conclusion that there is a universal diffusion of the nervous or sentient substance throughout all parts of the frame, certain organs possessing a greater proportion of it than others; and, as the modes of feeling of different parts are very various, there can be no doubt that the sentient power is modified, in different situations, by the manner and degree in which this substance is combined with other tissues. In the greater number of kinds of animals, and most distinguishably in the higher classes, are found filaments arising from all parts of their bodies, and which, combining into cords, or "nerves," proceed to combine themselves into masses, which, from their situation, have been called "central." This mode of arrangement it is which forms a nervous system; and it has for its end the influencing the state of each part, and of the individual, by the sensations of every other part, and also the production, in the central parts of the system, of certain qualities, between which and those of the other parts of the body there exists definite relations. In some animals of the lowest classes, a nervous system has not been discovered, and thence it has been inferred that these animals do not possess a nervous substance. But the inconclusiveness of this opinion appears from the consideration that the presence of a nervous substance implies that of a nervous system only in creatures possessing a number of distinct organs, and whose manner of existence requires that the sensations of those organs should be combined with each other; and from the fact that, in animals of the highest organization, the nervous substance is not distinguishable, in the simpler organs, from the tissues with which it is primarily combined. The nervous substance in the skin and mucous membranes of the human subject is not more easily recognized than in the body of a polypus; yet, that in those organs it exists in a state of continuous diffusion, there can be no doubt; and the perfect similarity of the functions of some of the lowest animals to those of certain organs in the highest,—the stomach and intestinal canal, for instance,—leads to the conclusion that the kind of organization which exists in the one exists also in the other. It is deserving of attention that, in the more highly organized animals, the nervous cords which conjoin the sentient extremities to the central masses of the nervous system form, on their passage, numerous connexions with each other, in some parts of the system assuming an intricate plexiform arrangement; while, in the lowest classes in which a nervous system is found, these collateral junctions are either wanting or exist in a very limited degree, the nerves proceeding directly from their origins to the central parts.

In the work the title of which is prefixed to this article, Dr. Philip applies to the treatment of certain obscure affections of the brain, upon which he supposes that many chronic diseases depend, those physiological principles upon the elucidation of which he has bestowed so much pains in his well-known "Experimental Enquiry into the Laws of the Vital Functions." The first two chapters of the four of which the Gulstonian Lectures consist are indeed little more than a repetition of that which Dr. Philip has already laid before the public eye so frequently, and in such a variety of forms; the title of the first chapter being "On the state of our knowledge respecting the General Laws of the Animal Economy at the time my investigations were begun," and that of the second, "The results of investigations respecting the General Laws of the Animal Economy."

In the former of these chapters, Dr. Philip considers the experiments and conclusions of M. Le Gallois; in the latter, he states the results of his own investigations, on which, he says, it appears to him, is founded the only correct view of the laws of our frame. From Le Gallois' experiments four principal conclusions had been drawn by that author, and by the committee appointed to report on his memoir. 1st. That the cause of all the motions of inspiration has its seat near that part of the medulla oblongata which gives rise to the nerves of the eighth pair. 2d. That the cause which animates each part of the body resides in the portion of spinal marrow from which the nerves of that part are derived. 3d. That, in like manner, it is from the spinal marrow that the heart derives its life and its powers; but from the whole spinal marrow, and not merely from any particular part of it. 4th. That the great sympathetic nerve takes its rise from the spinal marrow, and that the particular character of that nerve is to bring every part to which it is distributed under the immediate influence of the whole nervous power. On these conclusions of Le Gallois, Dr. Philip observes as follows:

"None of the experiments of M. Le Gallois, although at first view indicating that the spinal marrow possesses a power over the heart not possessed by the brain, when duly considered, afford this inference. . . . . . . His experiments demonstrate that, when a stilette of nearly the same diameter with the cavity of the spine is forcibly passed into it, . . . . . acting with such power on the spinal marrow as suddenly to destroy its mechanism, the power of the heart is instantly so impaired as to be incapable of maintaining the circulation: . . . on the other hand, . . . the brain may be wholly removed without at all influencing the power of that organ. . . . . It is not, on the one hand, a fair inference that, because the power of the heart was nearly destroyed by the injury done to the spinal marrow, it derives its power from that organ; because its power being derived from some other source, might be influenced by affections of the spinal marrow; nor, on the other hand, that the power of the heart being unimpaired by the removal of the brain affords any proof that its power may not be influenced by the brain while their nervous connexions remain. It was only necessary to reverse the circumstances of the experiment, in order to show the inaccuracy of the inferences of M. Le Gallois and the committee. This was done, . . . the spinal marrow was removed, and the brain crushed. The result also was thus reversed. In the former case, the power of the heart remained unaffected; in the latter, it was as much impaired as by the destruction of the spinal marrow." (P. 16.)

We agree with Dr. Philip that it is not fair to infer that, because the power of the heart is impaired by the injury done to the spinal marrow, it receives its power from that organ; and we extend this observation to the inverse experiment, which, we think, does not prove that the power of the heart is derived from the brain. In either case, we believe that the heart sympathised with a shock given to the system, as it does with the crushing of a limb, or with a strong impression made on the stomach; it being a law of the sympathy which exists in so marked a degree amongst the organs of the frame in the higher animals, that a violent injury, suddenly done to any part, affects the vocations and functions of all other parts for a period, and in a degree which depend upon their specific sensibility and their anatomical relations; while the same amount of injury, if inflicted gradually, produces comparatively trifling results. But we deny "that the brain may be wholly removed without at all influencing the power of the heart." It is true that, for a short time after the removal of the brain and also of the spinal marrow, the heart will continue to pulsate, and, by the aid of artificial respiration, its motions can be continued for a longer period; but these motions soon cease, not because the stimulus of red blood is withdrawn, (for it is not,) but because the relation which ordinarily exists between the heart and the central parts of the nervous system has been destroyed. It is a common error amongst physiologists, and one from which Dr. Philip is by no means free, to suppose that certain relations are not necessary to the ordinary functions of parts, because these functions do not cease immediately when those relations are suspended. Every organ in the body has what may be called a specific power, one which depends upon the organization of the part in which it inheres; and so far each organ may be regarded as having an independent existence: but in all animals in which there is a nervous system, properly so called, it is necessary to the permanence of the functions of organs that the relation which has been established, according to the kind of creature, between the separate parts and the central masses of the nervous system, should remain unaltered. There can be no doubt that certain states of the brain and spinal marrow, as well as of other parts of the body, influence the functions of the heart; while, at the same time, it is well known that the heart has a power of continuing its actions for some time after it has been removed from the body: and this power of independent existence seems to vary, in the different classes of animals, nearly in the inverse ratio of the organization of the creature.

Having combated the conclusion of Le Gallois, that the circulation in every part of the body depends on the corresponding portion of the spinal marrow, and having shown that the failure of the circulation which follows violent injury done to any part of the spinal marrow depends upon the impaired function of the heart, arising from the same circumstance, Dr. Philip goes on to say,

"The position of M. Le Gallois and the committee, that the great sympathetic nerve wholly derives its power from the spinal marrow, is a consequence of the opinion adopted by them respecting the exclusive powers of that organ, and must share the fate of the opinion on which it is founded; and the fallacy of the position that the functions of all the vital organs are equally dependent on the nervous system, appears from the simple experiment, . . . of interrupting the passage of the influence of the brain along the eighth pair of nerves, which uniformly destroys the functions both of the stomach and lungs, while it leaves the function of the heart wholly unaffected." (P. 20.)

The opinion that the sympathetic nerve wholly derives its power from the spinal marrow, is not to be defended, as there is reason to believe that the part of the nervous system to which the name of sympathetic has been given enjoys functions of its own, capable, no doubt, of being influenced by its relation to the other parts of the nervous system, to which it is connected by nervous filaments; but when Dr. Philip says, p. 41, "I think it will be admitted that its [the ganglionic system's] only functions are . . . . to combine and convey the influence of every part of the brain and spinal marrow," we must protest against being included in the number of those by whom such an admission is made. The part of the nervous system called sympathetic consists, as is well known, of nerves which, proceeding from various organs, combine with each other in forming small bodies called ganglions; from which circumstance this part of the nervous system has received (but, as we shall afterwards show, improperly,) the name of ganglionic. The structure of the ganglions is known to be a plexiform arrangement of nervous fibres, surrounded and supported by a peculiar substance of a darker colour. This mode of structure, and the relation which the sympathetic system bears to the organs from which its nerves proceed,—analogous to that between the organs and the simple nervous system of many of the lower animals,—led physiologists to the opinion that the ganglions of the sympathetic performed to the thoracic and abdominal viscera, and to some other parts, the functions of little brains, in which the sensations of the different parts were combined, and powers created of influencing the functions of the parts in connexion with them. We look upon this view of the functions of the sympathetic nerve as correct, and do not think that Dr. Philip's experiments tend, in the slightest degree, to impair it. He says, at page 37,

"The heart, the vessels, the stomach, the lungs, were all found to be under the influence of every part of the brain and spinal marrow. Even the action of the vessels, to their minutest ramifications, it was ascertained, by the aid of the microscope, could be increased, impaired, or even destroyed, by agents, the operation of which was confined to any portion of a certain extent of these organs; for the most powerful agent fails to influence either the vessels or any other vital organs, if confined to a minute portion either of the brain or spinal marrow."

Again, at page 38,

"While causes confined to either of them (the brain and spinal marrow,) were capable of increasing, impairing, and even of instantly, and almost wholly, destroying the power of the heart or capillary vessels, the total removal of either or both produced no sensible effect on them."

Now, these facts prove, what might have been expected, that a violent injury inflicted on many parts of the brain or spinal marrow is capable of influencing the functions of other parts of the frame; but the possibility of abstracting the brain and spinal marrow without sensibly, that is immediately, affecting the functions of the heart or capillary vessels, proves that not only the power of moving, but also of associating their movements, exists in those parts, for some time, even in the higher classes of animals, independently of the presence of the brain and spinal marrow. The consecutive or associated movements of the intestinal canal also are performed for some time after the removal of the brain and spinal marrow, and even with more activity after than before the removal of those parts; a fact of which Dr. Philip does not seem to have been aware.

The inference from these observations is, that, as the associated movements of parts in direct communication with the brain and spinal marrow is to be referred to their influence, so the analogous movements of parts in more immediate connexion with the ganglions depend upon the influence of these organs. This conclusion derives much strength from the consideration that the intimate structure of the brain and spinal marrow is of the ganglionic form. In the paper by Dr. Macartney already mentioned, we find it stated, at page 449 of the Report, that "by these means it will be observed [in the brain] that all the white substance, whether appearing in the form of bands, cords, or filaments, or simply pulp, is composed of still finer fibres, which have a plexiform arrangement, and that all these fibres, to the finest that can be seen, are sustained and clothed by a most delicate membrane. By the same mode of dissection, also, it is possible to make apparent the existence of still finer interwoven white fibres in all the coloured substances of the brain, in many of which the nervous filaments are so delicate and transparent that they are not visible until in some degree coagulated by the solution of alum, or by spirits. Dr. Macartney has thus been enabled to see twenty-six plexuses not hitherto described in the brain." And, at page 451, "the term plexus has been generally employed to signify an interweaving or crossing of filaments; but Dr. Macartney is satisfied that there is an actual union or intermixture of substance in both the plexuses of the brain and of other parts of the nervous system. He has discovered that the roots of the spinal nerves, instead of being connected with the medulla by mere contact or insertion, as hitherto supposed, actually enter into the composition of the filaments of the spinal marrow, and that these roots of nerves (as they are called,) form communications with each other within the substance of the medulla. With regard to the cerebral nerves, also, it can be shewn that they are continuous with the cerebral plexuses in their immediate neighbourhood." At page 453, speaking of the coloured substance, he says, "One obvious purpose of its existence is to give support and security to the finest subdivisions of the sentient substance; we therefore find that it affords such protection in proportion to the necessity: hence, in the brain the coloured substance is soft and tender, while in the ganglia of the nerves it is generally dense and firm. Besides, however, forming a nidus for the ultimate plexuses of the sentient matter, the coloured substance would seem to fulfil some other use not yet ascertained." It would seem that the coloured substance, which appears to possess a peculiar vascular arrangement, is capable, by its relation to the sentient substance, of modifying the qualities of the latter. There can be no doubt that the peculiar properties of highly organized substances are modified, in many instances, by changes in their relations too trifling to influence their common qualities. These observations shew that the distinctive term "ganglionic" has been without sufficient reason applied to the sympathetic nerves, and lead irresistibly to the conclusion that the peculiar structure which, in the brain, is connected with the existence of certain qualities or powers, subserves analogous functions in all other parts of the nervous system in which it is found to exist.

Regarding the "position that the functions of all the vital organs are equally dependent on the nervous system," we have to remark the laxity with which the term "nervous system" is employed by Dr. Philip. In the passage just quoted, he employs it to express merely one of the central parts of that system, as appears from the experiment to which he alludes, in which the division of the eighth pair of nerves destroyed the functions both of the stomach and lungs, while it left the functions of the heart unaffected. The nervous system comprises not only the brain, spinal marrow, other ganglia, and nervous cords, but every particle of nervous substance existing in organs, in the most subdivided form. Legallois is right in saying that the functions of all vital organs are equally dependent on the nervous system, in as much as the functions of each and all of them are in absolute dependence on that system. The presence of venous substance in an organ is as necessary to the performance of its functions, as is the presence of any other tissue. What Dr. Philip's experiment proves is, that the functions of all the vital organs are not in the same relation to the functions of all the parts of the nervous system; than which position nothing can be more true.

Dr. Philip advocates the theory that an influence, derived from the brain and spinal marrow, is the cause of secretion and the nutritive functions; and supposes that the influence is the galvanic fluid, collected or produced in these organs and transmitted along the nerves. Dr. Alison* and Dr. Henry† contend, on the other hand, that the processes above mentioned "are independent of any influence or energy necessarily derived from the nervous system;" meaning by that, the central parts of the nervous system. Dr. Alison explains the cessation of digestion on cutting the eighth pair of nerves, by supposing that thus the sensibility of the stomach was destroyed, and that cessation of function was the result of impaired sensibility. In reply, Dr. Philip says,

"In these experiments the sensitive functions remained uninfluenced. When the secretion of gastric juice was wholly destroyed by dividing and separating the divided ends of the eighth pair of nerves, the appetite, as long as there was any part of the gastric juice, previously in the stomach, which remained disengaged, was as good, and the desire to breathe as great, after as before the operation. There was no symptom whatever of the impaired sensibility supposed by Dr. Alison. The assimilating functions alone were impaired or destroyed, precisely according to the degree in which their organs were deprived of nervous influence, proving that the brain and spinal marrow include the organs on which these functions depend, as well as those of the sensitive system." (P. 29.)

Dr. Philip has here, we conceive, fallen into two very considerable errors: he imagines that the appetite for food is produced by the presence of gastric juice in the stomach, and he confounds individual with organic sensibility. That the desire for food does not depend on the presence of the gastric fluid is proved by many facts: for instance, the ravenous craving for food in certain cases of diseased mesenteric glands, and in the recovery from fevers; in both which cases the desire for food is proportioned to the want of nutrition in the system, and by no means to the quantity of gastric juice secreted, or to the digestive powers of the stomach. Birds are satisfied when they have filled their crops with food, although the gastric juice is not secreted in those organs, and is not brought into contact with the food until some time after the appetite has been appeased. What will Dr. Philip say to the fact that the green grasshopper has been fed with his own stomach, taken out of his

* Dissertation on the state of Medical Science from the termination of the 18th century to the present time, in the Cyclopædia of Practical Medicine.

† Report of the British Association for the advancement of Science, 1833.

body, cut into pieces, and presented to the animal, by which it was greedily devoured? But why, it may be asked, does an animal, in which the eighth pair of nerves has been cut while the stomach is empty, begin to eat immediately after, and continue to eat until its stomach is gorged with food? We reply, that it begins to eat because it is hungry; and it knows not when to stop, because that state of the stomach on which satisfaction of appetite depends is, in consequence of the cutting of the eighth pair of nerves, not felt by the individual. Dr. Philip infers, from the disposition to continue to eat, that the sensibility of the stomach was not impaired; we infer from it, that the individual was not sensible of the state of the stomach. But, if the continuity of the eighth pair is necessary to apprize the individual of his relation to his stomach, it is not less so to the perception, by the stomach, of its own relation to the individual. We have already stated our opinion that permanency of function in parts requires a maintenance of their ordinary relations to the nervous cavities; and we have no doubt that the perception of these relations by the organs between which they are naturally established is essential to the due performance of their functions. We are thus led to the conclusion that digestion ceases after the cutting of the eighth pair of nerves, not because the access of the galvanic or any other fluid is thus prevented, but because an essential relation of the stomach has been destroyed.

But Dr. Philip restored the function of the stomach by the application of galvanism. On this we observe, that it is possible, in many instances, where the ordinary relations of parts have been destroyed, to induce by the establishing of new relations a temporary continuation of function. Many substances, called "stimulants," are capable of exciting motion, or other functions, in living parts, even when separated from the body; but such capability does not prove the identity of any of these substances with the cause of function in the living body; for, if it proves the identity of one of them, it proves the identity of all. The sense of fear produces, in many persons, the same effect upon the lower intestine as a purgative medicine; we cannot thus infer that fear and purgatives are identical. Galvanism is a substance capable of exciting the specific actions of many parts of the body in a greater degree than any other substance hitherto known; and it is just as reasonable a supposition that secretion should, for a time, be induced in the living stomach by the excitement of its sensibility by galvanism, as that motion should be produced in a muscle by the prick of a pin or immersion in warm water. It is as easy to explain the connexion between sensibility and secretion as between sensibility and motion. The functions of secretion have been performed in the human fœtus in many instances in which the brain and spinal marrow were wanting; and that secretion, taken generally, is independent of a nervous system, is proved by the mode of nutrition in the simplest animals and plants; but we agree with Dr. Philip that, in the higher classes of animals, after birth, the due performance of the secretions is influenced by the functions of the brain and spinal marrow, as well as by the state of other parts of the system, although we do not believe "that in the brain and spinal marrow reside the organs of assimilation," nor do we admit Dr. Philip's explanation of the fact.

"There is still another subject to be considered before we can obtain a correct view of the influence of the nervous system in determining the nature and progress of disease. I refer to that effect on each other of the various parts of our frame, to which the term sympathy has been applied, and by which the progress of diseases, and consequently their nature, particularly as regards the more protracted cases, are more extensively influenced than by any other cause.

"It is necessary, in the first place, clearly to determine what we mean by sympathy, or, I should rather say, to point out the sense in which I shall employ it; for few terms have been employed with less precision. We do not refer to what is called sympathy all the effects of distant parts on each other, although there are few of these which have not, by some writer or other, been referred to it. I shall not refer to sympathy . . . any instances in which distant parts influence each other, where the structure of our bodies at once points out the channels of communication. But when a cause, for example, which makes its impression on the stomach, produces palpitation, I shall regard it as affecting the heart by sympathy, because it at once appears from the structure that there is no direct channel of communication between apparently the only parts concerned. The term sympathy, then, may be defined the influence of distant parts on each other, between which the mere structure of our bodies, compared with the phenomena, do (does) not point out the exact channels of communication." (P. 54.)

Now, we are of opinion that, the more we know of the structure of bodies, the better shall we be enabled to recognize and explain the various sympathies between their parts. But Dr. Philip says, p. 56, "I shall not refer to sympathy the influence on each other of the parts concerned in any act of volition." Yet the mere structure of our bodies does not more satisfactorily point out the channel of communication between the brain and a voluntary muscle than between the heart and stomach. The communication between the heart and stomach is not so direct as that between the brain and muscle, but it is equally manifest. Dr. Philip says, p. 58, "the various parts of the living animal may be divided into active and passive. The belly of a muscle is the active, the tendon the passive part. In like manner, the brain and spinal marrow are the active, the nerves the passive part of the nervous system; the latter possessing no power but that which they derive from the former." And, at p. 78, "the brain and spinal marrow are the only active parts of the nervous system, all changes in the system originating in them." We dissent from this view of the matter, and think that the sentient extremities of nerves are as active in recognizing the qualities of substances, and the nervous cords communicating sensations, as are the brain and spinal marrow in discharging their peculiar functions. And that many, indeed almost all, changes in the nervous system originate in alterations of the sensibility of distant parts, many of the phenomena of disease, and amongst others the effect of certain poisons on the animal frame abundantly testify.

Dr. Philip further says, p. 59, "the phenomena of sympathy take place through the active parts of the nervous system, and consequently . . . depend on organs which belong to the central parts of that system;" and rejects the idea that parts can sympathise with each other through the medium of nervous communications, by the ganglions of the sympathetic, or otherwise. Although association of movements to a certain extent seems to exist in some plants, and in the lowest classes of animals, without the intervention of a nervous system, the presence of a nervous system seems to be necessary to the sympathies of disease; and, in proportion to the complication and number of parts of which this system consists, will be found the extent, and the influence of sympathy on the functions of organs. But there are many reasons why the sympathies of parts should not be referred exclusively to the influence of the brain and spinal marrow; the structure of ganglions, wherever they are found, and the relation which they bear to the parts from which they receive nerves, point them out as a medium of sympathy; and the peculiar course and combinations of certain nerves, as of the recurrent branches of the eighth pair, the pterygoid nerves, the chorda tympani, as well as the intricate plexuses formed by the nerves of the thoracic and of the abdominal viscera, and of some other parts, indicate that these more direct communications between parts also may influence their functions. Dr. Philip overlooks what we regard as a most important distinction, namely, that the brain and spinal marrow are the chief medium of those sympathies which produce acts or sensations of the individual, while the other nervous media are subservient to those sympathies not necessarily connected with individual consciousness or volition. He indeed admits two media of sympathy, one belonging to the vital, the other to the sensitive organs, but he places both these media, or "centres of sympathy," in the brain and spinal marrow. He says,

"The parts of the brain and spinal marrow associated with the organs of the sensitive system, and those associated with the organs of the vital system, are distinct sets of organs, having different localities, and obeying different laws. It is quite evident, therefore, that if both the sensitive and (the) vital functions in various parts of our frame sympathise, it cannot be through the same parts of the brain and spinal marrow; as these functions depend on different sets of organs, their centres of sympathy must be different." (P. 62.)

The supposition that the parts of the brain and spinal marrow, associated respectively with the vital and with the sensitive organs, are distinct from each other, is inconsistent with anatomical facts; as the spinal nerves are the means of connexion to the spinal marrow of the various ganglia and plexuses belonging to the abdominal and thoracic viscera, and the eighth pair of nerves and sympathetic, which connect the stomach, lungs, and heart to the brain, are conjoined to it in parts common to these nerves, and others subservient to sensitive functions. The ordinary sensations of vital parts are not recognized by the individual, although there can be no doubt that they are by the parts themselves, and serve to modify each other's functions; but any strong impression on a vital part, or increase of its sensibility, is made known to the individual in a manner analogous to that by which he becomes acquainted with the properties of the external world. Certain states of the vital organs are capable of modifying the sensations of the individual, although he is not always able to refer the alteration in his feelings to the part from which that alteration is derived; and, on the other hand, changes in the state of feeling in the individual influence the sensations and functions of vital organs.

Dr. Philip proceeds to illustrate his views of sympathy by comparing those of the stomach and liver. He says,

"There is no organ whose sympathies are absolutely confined either to the sensitive or (the) vital system; all organs more or less partaking of the functions of both: but that the different species of sympathy prevail most in different organs, a thousand phenomena assure us . . . . . In no other organ are the sympathies of the sensitive system so powerful as in the stomach; an organ of the most acute sensibility; but the sympathies of the vital system are much more powerful in the liver, which, although of very dull feeling, influences, and is influenced by, the vital functions of distant parts, more powerfully (if we except the brain itself,) than any other organ." (P. 64.)

Dr. Philip considers "that between the brain and the liver there is a sympathy which does not exist between the former and any other organ, for which no anatomical investigations would prepare us. Melancholy even derives its name from affections of the liver. Hence also the sick headachs, and other affections of the head which so generally attend what are called bilious complaints." From the sympathy which exists between the liver and brain, and between the latter and other vital organs, Dr. Philip endeavours to shew that organic disease of the brain may arise from affections of the liver, and, by a reflex operation, disturbance of the secretory and other functions may follow upon the state of the brain thus produced: he also thinks that disease of the liver, producing, by sympathy, defective secretion and assimilation, may thus impair the state of the brain, which organ, reacting on the parts originally affected, will aggravate the symptoms of disease.

For the details, we must, however, refer our readers to the work itself, and proceed to the practical part of our subject. At page 79, Dr. Philip says, "As we have seen that on an agent" (the galvanic fluid) "supplied by the brain and spinal marrow, the functions of assimilation, the most important of the animal economy, depend; it necessarily follows that the derangements to which their immediate organs are subject may be of two kinds; they may either be the effect of causes acting directly on the organs themselves, or on those organs which supply an agent essential to their functions." The diseases arising from the latter cause are those which Dr. Philip proposes to consider, and he selects the affections of the digestive organs to illustrate his principles, as those organs are "both of the most powerful and extensive sympathies, and those the functions of which are most easily made the subject of observation." Having alluded to the effects of the mental functions on those of assimilation, he says, p. 81, "We find similar effects from diseases or accidents affecting any considerable portion of the brain or spinal marrow;" and expresses his surprise that "it had not occurred to physicians that, in cases of chronic derangement of the assimilating functions, as in more acute affections of the brain and spinal marrow, the fault might sometimes be in those organs." In some cases of indigestion which had come under his notice, he says, page 82,

"Death seemed to arise from the failure of the digestive process alone; there was no prominent symptom that was not referrible to its organs, and the patient, emaciated to the last degree, appeared to die of inanition, in consequence of these organs, even where food could still be taken, being incapable of effecting the necessary changes on it. It was in considering these cases, . . . . that I was led to suspect that the fault might be in the central parts of the nervous system; and, on examining the bodies of those who died in this way, I found the brain diseased, and particularly in the parts towards its base and the medulla oblongata, from which the vital nerves proceed . . . . . " p. 83. "I need not say that it is of essential consequence to be able to distinguish these cases from those of ordinary indigestion at an early period,—the only period at which there is any hope of arresting their fatal course . . . . I shall, in the first place, point out the best diagnosis at which I have been able to arrive; for it will readily be perceived, from what has been said, by those acquainted with the principles of our profession, that there must be great difficulty in such a diagnosis."

We refer our readers for the method of forming it to Dr. Philip's work, from page 83 to page 89 inclusive, from which however we shall make the following extract.

"When the patient is not of a variable and hysterical habit,—when the occasional causes have been of a serious and permanent nature, and the nervous symptoms have not shewn themselves for some time after the first application of such causes,—when there is not such derangement in the digestive or other organs chiefly affected, as accounts for the severity of the nervous symptoms,—when the affections, both of mind and body, are less variable than is usual in what are called nervous complaints, and particularly apt to be referred to the same parts of the body,—when there is constantly a more or less general tendency to derangement in the secreting system,—when the heart is more irritable and the lungs less free, the nervous symptoms not yielding so readily as usual, the depression of spirits more uniform, and the pulse tighter than we should expect to find it from the other symptoms,—when either the recurrence of feverishness or a sense of chilliness and debility is more frequent than is usual in nervous complaints,—when the constitution seems more affected than usual by the continuance of the disease, the strength on the whole decaying,—and particularly when the countenance assumes a sallow colour and an habitually irritable and anxious expression; when the usual means are not attended with their usual effects, our stomachic medicines being in a great degree powerless, and alteratives producing but a transitory, if any, improvement in the abdominal secretions; when these, or several of these circumstances, are well marked in what are called nervous complaints, I have been assured, by repeated observation, that they are not to be safely disregarded." (P. 86.)

The catalogue of symptoms given above, is of a most melancholy description, and ought not by any means to be disregarded; but we are quite sure that they might arise from causes in which organic affections of the brain or spinal marrow had no share.

We shall now turn to the cases. The first is that of Mr. A., an Oxford student, whose case was regarded, by all persons but Dr. Philip, as one of common indigestion. Not improving under the Oxford physicians, he was brought to London, and placed under the care of two well-known physicians. In a few weeks Dr. Philip was called in; expressed his fears of a fatal termination, and stated his opinion that, although the stomach and duodenum were the organs most prominently effected, the origin of the disease would be found in the brain. The patient died in a fortnight, apparently from inanition. The body was examined about twenty-four hours after death.

"On opening the cavity of the cranium, the membranes and brain were found tolerably healthy, perhaps rather softer than usual, particularly as regards the cerebellum and base of the brain, which, together with the medulla oblongata and cerebral nerves, appeared reduced to a pulpy state; so much so, that they would not bear the slightest handling. The viscera in the cavity of the chest presented no unusual appearances; the stomach larger than usual, from distention, . . . . the pylorus more vascular than usual, and the duodenum much more dilated, vascular, and attenuated than is natural. The whole of the small intestines were . . . gorged with blood, and of a very dark colour. The liver, spleen, kidneys, and pancreas, were healthy." (P. 91.)

We cannot but be struck with the inconsistency of this report of the examination; in which it is stated that the brain was *tolerably healthy*, and yet, that the *cerebellum, base of the brain, medulla oblongata, and cerebral nerves, were reduced to a pulpy state, and would not bear the slightest handling!* What warrant have we, or has Dr. Philip, (who, we conclude, was not present at the examination,) that "the liver, spleen, kidneys, and pancreas, were healthy!" But the extraordinary feature of this case is, that, although certain most important parts of the cerebral mass were in a state of disorganization, the functions to which they were subservient do not appear to have suffered in the smallest degree. The senses of smell, sight, taste, and hearing, the sensations and motions of the face, and indeed, as far as we are told, all the functions but those of digestion remained unimpaired; while the cerebral nerves, medulla oblongata, and cerebellum, which, it is generally admitted, are connected with those functions, were reduced to a pulp. "The viscera in the cavity of the chest presented no unusual appearances," although the medulla oblongata had been unfitted by the abolition of its structure, for supplying to those organs that "agent on which their functions depend;" and the withdrawing of which for a few hours, we are told, produces, in them, serious organic disease. Either the morbid appearances in this case have been incorrectly stated, or they are fatal to Dr. Philip's conclusions.

"The next case is that of Miss C., which run (ran) the same course as the preceding, but was of longer duration, having been protracted for more than two years; and here also the patient appeared to die of inanition."

"The following is Mr. Earle's account of the appearances:—'In the head, slight effusion beneath the arachnoid membrane; substance of the brain very soft, particularly the crura cerebri, and upper part of the pons varolii, which was quite pulpy. Blood-vessels in the substance of the brain large, and loaded with blood. In the chest, *right lung greatly compressed by the narrowness of the inferior margin of the ribs, from old adhesions between the pleura costalis and pulmonalis.* Substance of the lungs firm and hepatized. Left lung more healthy than the right, but slightly hepatized at its upper part.' This state of the lungs, it may be remarked, is peculiarly characteristic of a failure of nervous influence, as appears from those experiments in which the influence of the brain was prevented from reaching the lungs."

Of this, the preceding case was not, it must be confessed, a striking example. "The patient had been subject to cough and oppressed breathing; pulmonary symptoms, however, had never been a prominent part of the disease."

"'The heart,' Mr. Earle proceeds, 'was remarkably small. In the pericardium, about two ounces of water. In the abdomen, *stomach and duodenum much displaced by the compression of the chest by the stays.* Towards the pylorus, the stomach much thickened and indurated, the pylorus hard and contracted. The duodenum large and flaccid; the mucous surface very vascular, villous and soft, readily breaking down on the slightest touch, and apparently approaching to a state of ulceration. Substance of liver hardened. Spleen and kidneys . . . healthy. Intestines congested.'" (P. 92.)

In this case, the passages in *italics* seem to us to point out the causes of disease. There had been old disease of the pulmonary organs, and the compression of the abdomen by stays, had displaced, and probably induced disease in, the stomach and duodenum. The disease in the liver may have been sympathetic with that of the other viscera of the abdomen, or may have been induced by the same cause. Indeed, we look upon the practice of wearing tight stays as one of the most fruitful sources of disordered sensations, and ultimately of visceral disease and deformity. There is not, in the two cases detailed, any thing which induces us to suppose that the cerebral disease had necessarily preceded the alterations in the thoracic or abdominal disease; and the manifest disproportion which the affections of the latter bear to those of the brain in the respective cases would lead to the opposite conclusion. We have little doubt that many appearances, described as morbid, in post-mortem examinations, are the result of decomposition after death; and, in the cases above mentioned, the absence of disturbance of the ordinary cerebral functions during life would lead us to suppose that such an error might have crept into the investigation.

We must pass over the remaining statements and reasonings of Dr. Philip on this subject, and apply our observations to the treatment recommended.

Dr. Philip says, p. 107, that

"All agents capable of effecting the moving powers of living animals may act either as stimulant or sedative, . . . . according to the degree in which they are applied, the stimulant arising from the less, the sedative from the greater, application of them."—(P. 109:) "The assimilating functions depend on the . . . . powers . . . of the nervous and muscular systems . . . ; disease always more or less consists . . . . in a failure of one or both of these powers . . . . . . It is therefore, more or less, in all diseases, the object of the physician . . . . to restore them. By the stimulant, that is, the invigorating effect of his means, this is accomplished."—(P. 110:) "It is in vain to endeavour to restore the digestive organs, . . . . by means directed to them alone, when the cause of failure is in a distant part. The only means which can succeed must include those which give vigour to the central parts of the nervous system, in which the immediate cause exists."—(P. 114:) "In the cure of all diseases the object is to restore the healthy functions of the parts concerned; and, it is only in proportion as this is effected, that the tendency to disease of structure is corrected."—(P. 116:) "It is here necessary, therefore, as far as possible, to remove all causes tending to disturb the functions either of mind or body . . . . It is necessary, as far as we can, to divert the patient's mind from his sufferings by change of scene and such occupations as amuse without fatiguing; and, if we succeed in restoring the healthy state of the mental functions, a great step is made towards the restoration of all the others."

It is consolatory to us to bear our testimony to the excellence of the precepts contained in the last two sentences of our quotation. In a great number of cases of disease of the digestive organs, accompanied by morbid feelings, change of scene and the exciting of pleasurable sensations are among the most effectual remedies; and, as such, are sought after and employed by all sorts and conditions of men. But the influence of mental feelings on diseased sensations is not a proof that those sensations depend upon changes in the structure of the brain. We have known long-continued mental anxiety and discontent produce fatal disease of the stomach and pylorus; while, at the same time, all the mental faculties, and the functions of the lungs, remained unimpaired. Such cases appear to us to resemble those in which affections of the mind increase, alter, or suspend particular secretions; but do not seem to depend upon any organic change in the substance of the brain. Organic cerebral disease is attended, more or less, by other symptoms than those of impaired pulmonary or digestive function: in such cases we commonly find that the intellectual, perceptive, or voluntary powers are affected in a greater or less degree, and painful sensations are experienced in or near the seat of the disease. In those cases of disease of the spinal marrow, which Dr. Philip considers analogous to the affections of the brain of which we are speaking, besides impaired vital functions, pain and want of voluntary power are constant attendants.

At page 127, Dr. Philip says,

"With regard to the effects of local measures, directed to the brain itself, the result of my experience is, that unless a greater than usual determination of blood to this organ has taken place, they are less beneficial than in any other local disease; and, although I was not prepared to find this so much the case as it is, when we consider that the state of the brain is influenced by, as well as influences, every other part, it is a result for which, in its more chronic affections, we might be prepared."

We are disposed to explain the failure of local remedies in these cases by the supposition that the symptoms of disease do not depend on organic affection of the brain. In organic disease of the spinal marrow, which is attended by peculiar symptoms mentioned before, and which is generally preceded by disease of the adjacent bones or cartilages, local treatment is efficacious.

"The means chiefly directed to the mind, however, if trusted to alone, will generally fail, except in the most favorable cases, and where they can be employed to the greatest extent; and we should have little hope of frequent success, if we were not possessed of others both more powerful, and more under command." These are mercury and antimony, . . . . medicines, in large doses capable of the most powerful effects on individual parts, and, in small doses, of the most gentle and salutary effects on the whole system." (P. 116.)

Dr. Philip believes that a tendency to inflammation in any part depends upon a debilitated state of its capillary vessels; and regards stimulant remedies as those applicable to such a state of disease. He says (p. 122,) "of all the medicines we possess, mercury and antimony are the most powerful in exciting the extreme vessels, on the debility of which we have seen the tendency of functional derangement to terminate in disease of structure depends." Although we do not agree with Dr. Philip that inflammation depends on weakness of the capillaries, or that stimulant treatment is in most cases to be employed in inflammation, we do agree with him that mercury given in small doses has a powerful effect on many forms of disease. We think that this mode of administering mercury is, however, more applicable to cases of diseased function, than to those in which there is evidence of actual inflammation. In the latter cases, the speedy affection of the system by the medicine appears to be one of the most efficacious means of control. We do not pretend to explain the influence of mercury on the living system; but its power of increasing many of the secretions is that property by which it immediately recommends itself to our notice, and, to which its influence or functional diseases may be very probably referred. In the greater number of affections of the assimilating functions, attended by what are called nervous symptoms, we have no doubt that the latter arise, not from any local affection of the brain or spinal marrow, but from the perception, by the individual, of the unusual and morbid sensations of the organs manifestly affected with disease. There can be no doubt that continued disease in the organs of assimilation frequently produces organic affection of the central parts of the nervous system; but, when this effect has been established in the brain or spinal marrow, we think it is accompanied by new symptoms which do not fail to point out the nature of the affection. Thus, while we dissent from Dr. Philip's view of the relation between the state of the assimilating organs and that of the brain, in the diseases of which we are now treating, we approve, on general principles, of his method of treatment in those cases which do not yield to the simpler remedies first suggested by him. It is now generally known that mercury, in small doses, is capable of producing most of its specific effects, without inducing the debility attendant on its administration in larger quantities; and to this point Dr. Philip directs special attention. Antimony, from its influence on the cutaneous functions, is a useful adjunct to the mercurial treatment; and, p. 123,

"In those affections of the head itself, which frequently attend such cases, particularly a sense of tightness and other inflammatory symptoms referred to the head, and depending on the determination of blood to it being increased by the obstructed state of the digestive organs, considerable advantage is often derived from combining with the other means the tartrate of antimony, and occasionally increasing it till some degree of nausea is produced."

This we might expect. In "occasional attacks of extreme nervous irritation," Dr. P. praises the 'soothing effect of a combination of tartrate of antimony and henbane, . . . . two or three grains of the latter, and as much of the former as can be borne without nausea, generally from the eighth to the fourth part of a grain." From this dose, repeated every half hour until nausea comes on, "composure may generally be obtained, even in maniacal cases." Dr. P. recommends, as a "medicine which excites the nervous with the least disturbance to the sanguiferous system," the carbonate of ammonia: "but, in many instances, even the mildest stimulants increase the tendency to heat and restlessness." Saline medicines are found to be necessary to allay inflammatory tendencies, and to these are added local means for the relief of particular symptoms.

Dr. P. finally recommends voltaic electricity, as a substitute for the nervous influence, and states that, in the earlier stages of disease, he "has repeatedly seen it successful where all other means had failed." In support of his views on this subject, he introduces a letter from Mr. Earle to him, dated 1822, and already published in the author's "Experimental Enquiries," and also in the "Journal of the Royal Institution," in which letter it is recorded that three cases of dyspnœa and indigestion, arising from disease of the spinal marrow, had been benefited by the application of galvanism. We regret that our experience has not been so satisfactory: we have not been able to ascertain, in more than one case, and which was believed to be impaired function of the liver, that galvanism has been productive of beneficial effects. In some cases in which it has been applied, we have been informed that it seemed to aggravate the symptoms; and in the greater number its effects are not perceptible. The low degree of estimation in which galvanism stands as a remedy with the more experienced members of the medical profession, is a convincing proof of its inefficacy.

The work under our consideration has occupied more of our time than seems due to the number of its pages. But it is, in fact, a synopsis of the greater part of the author's previous publications, and involves a consideration of his own physiological opinions and of those of his antagonists. We cannot commend its style, which is diffuse and unconnected. The structure of the sentences is often faulty, abounding in grammatical errors, and obscuring the meaning in a multiplicity of words. We entertain an unfeigned respect for a physician who has devoted so much of the leisure left him, after the toils and anxieties of a large practice, to physiological investigations bearing upon pathology. In investigations attended with so many difficulties, some errors are almost unavoidable, and are not proper objects of critical severity. We can even pardon Dr. Philip's very numerous repetitions of himself. But we have thought it our duty freely to examine the results of his labours, and as freely to discuss their practical applications.

---

